# Genetic Disorders Detectable by Fetal MRI: A Review

**DOI:** 10.3390/diagnostics16071112

**Published:** 2026-04-07

**Authors:** Kwok Chun Wong, Tsz Ho Chow, Claudia Cheung, Joyce Pui Kwan Chan, Jonan Chun Yin Lee, Elaine Yee Ling Kan, Winnie Chiu Wing Chu

**Affiliations:** 1 Department of Radiology, Hong Kong Children’s Hospital, Kowloon Bay, Hong Kong; 2 Department of Imaging and Interventional Radiology, Prince of Wales Hospital, Shatin, Hong Kong; 3 Department of Diagnostic and Interventional Radiology, Queen Elizabeth Hospital, Jordan, Hong Kong; 4 Department of Imaging and Interventional Radiology, Faculty of Medicine, The Chinese University of Hong Kong, Shatin, Hong Kong

**Keywords:** fetal MRI, genetic disorders, genetics, chromosomal abnormalities, prenatal diagnosis, congenital anomalies, congenital malformations

## Abstract

Fetal MRI has been increasingly used in diagnosis and assessment of congenital anomalies and conditions by providing detailed structural information. However, such information is only part of the whole landscape of these genetic disorders. Given that genetic disorders are associated with significant morbidity and mortality in infants, multidisciplinary team management is essential for perinatal management and parental counseling. In the past two decades, there are advances in both fetal MRI and genetic testing for prenatal diagnosis of genetic disorders. This narrative review consolidates the current literature and aims to provide a systematic overview of fetal MRI applications in genetic disorders affecting the central nervous system, craniofacial structures, skeletal system, lungs, and urinary system. Understanding embryological and genetic basis as well as imaging phenotypes of genetic disorders are important in improving perinatal diagnosis and management.

## 1. Introduction

Genetic disorders affecting multiple organ systems are associated with significant morbidity and mortality in infants [1,2]. Globally, according to WHO, it is estimated that about 6% of total births annually occur with a defect of genetic or partially genetic origin [3]. Fetal MRI is a well-established prenatal diagnostic radiological investigation for congenital anomalies and conditions of brain, spine, head and neck, chest, congenital heart disease, skeletal dysplasia, and genitourinary disorders. A review article by Hengstschläger in 2006 provided an insight into how an efficient interaction between genetic testing and fetal MRI to improve prenatal diagnosis and parental counseling [4]. And in the last twenty years, literature on fetal MRI had shown an improved diagnostic accuracy than ultrasound alone in non-CNS congenital anomalies of fetal body and detection of fetal brain abnormalities [5,6]. With advancement in prenatal genetics and genomics analysis, assessment of fetal health conditions today requires integrated understanding of imaging findings with an underlying genetic basis, embryology, and pathophysiological mechanism. However, radiology and genetics literature often address same conditions in different perspectives. For example, radiologists focus on detection of macroscopic cysts on imaging when differentiating autosomal dominant polycystic kidney disease and autosomal recessive polycystic kidney disease, while geneticists focus on the different gene mutations and inheritance patterns for parental counseling. The purpose of fetal MRI in general is to complement ultrasound examination by confirming the ultrasound findings or providing additional information for a specific clinical question [7]. Currently, both the American College of Radiology (ACR) and the Society of Pediatric Radiology (SPR), as well as the European Society of Pediatric Radiology (ESPR) Fetal Task Force, issued recommendations on indications for fetal MRI in the central nervous system and extra-central nervous system. With the exceptions of family history of severe brain diseases or high risk of development of brain abnormalities such as fetal infection or stressful maternal environment, most indications are anomalies or malformations in different organ systems suspected or not adequately assessed by ultrasound [8,9,10]. While disease-specific reviews and literature are available, there are few comprehensive reviews addressing fetal MRI applications across multiple organ systems in genetic disorders. This narrative review aims to provide a systematic overview of fetal MRI applications in genetic disorders affecting the central nervous system, craniofacial structures, skeletal system, lungs, and urinary system. Relevant publications focusing on fetal MRI diagnosis of genetic disorders were identified though PubMed, Embase, and Google Scholar using keywords related to the topic, including fetal MRI, genetics, genomics, prenatal diagnosis, chromosomal abnormalities, brain malformations, mitochondrial disorders, craniofacial syndromes, eye syndromes, skeletal dysplasia, congenital lung malformations, urinary system genetic disorders, and congenital anomalies of the kidney and urinary tract. Search period was within the last 20 years. Several older references were used for historical information. By presenting information according to embryological principles and genetic mechanisms, we provide a framework for clinicians from different disciplines, including maternal–fetal medicine specialists, pathologists, radiologists, geneticists, and pediatricians for accurate diagnosis, perinatal care, prognostication, and parental counseling.

## 2. Central Nervous System Disorders

### 2.1. Brain Malformations

**Figure 1:**
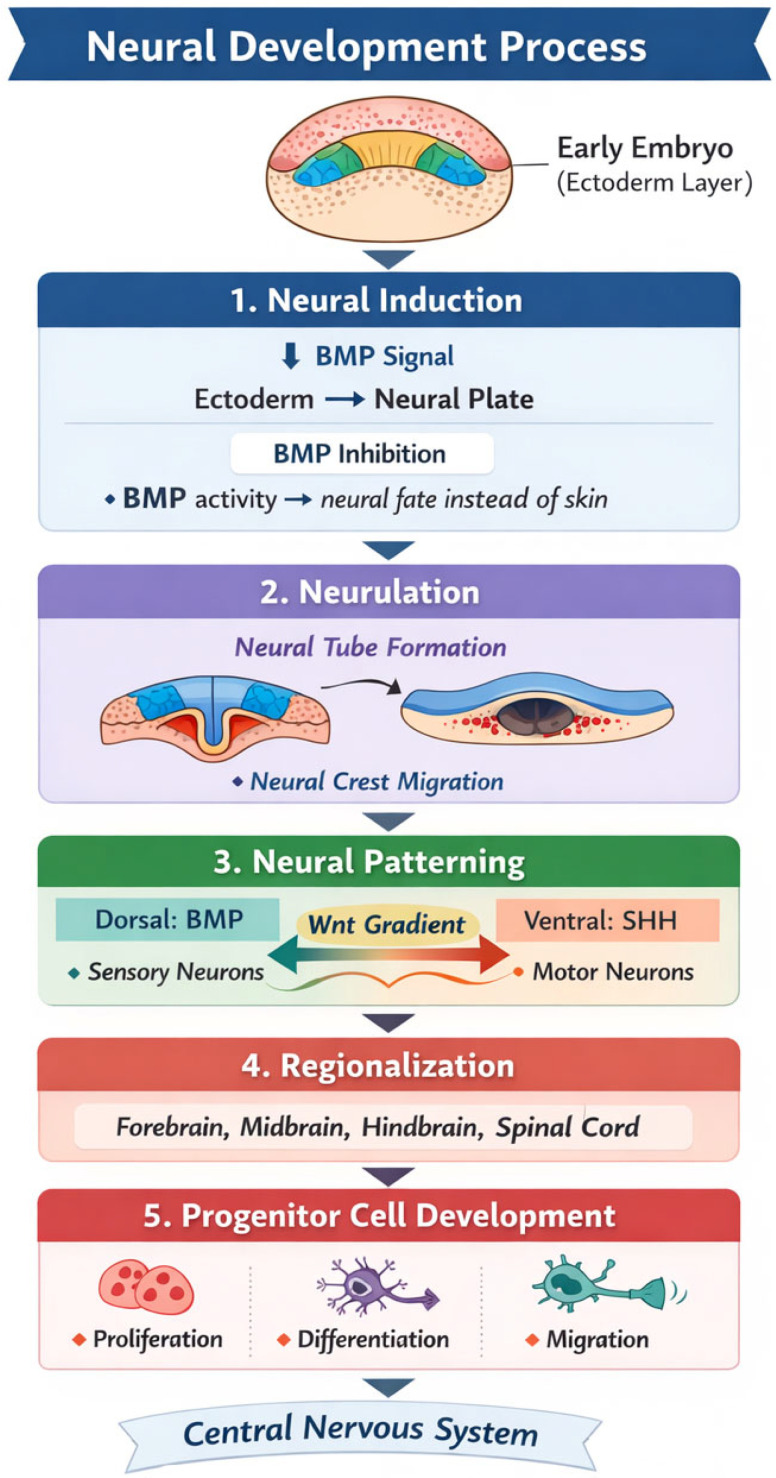
Diagram illustrating the stages of neural development and its key signaling pathways.

Central nervous system (CNS) development involves a series of complex processes, starting from the formation of the neural tube, which subsequently differentiates into the brain and spinal cord. This complex process is regulated by key signaling pathways such as Sonic Hedgehog (SHH), Wnt, and BMP, which guide the proliferation, differentiation, and migration of neural progenitor cells (Figure 1) [11,12].

The genetic basis of CNS development is equally intricate, involving numerous genes that regulate these signaling pathways. Mutations in these genes can lead to various CNS malformations. For example, mutations in the LIS1 gene cause lissencephaly by impairing neuronal migration; mutations in the DCX gene result in subcortical band heterotopia; mutations in the TUBA1A gene leads to a spectrum of lissencephaly disorders by affecting microtubule function; and mutations in the KIF7 and GLI3 genes lead to central nervous system malformations such as acrocallosal syndrome (ACS) by disrupting the Sonic Hedgehog (SHH) signaling pathway [13,14].

Acrocallosal syndrome (ACS) is a useful example for illustrating this developmental process. ACS is an autosomal recessive disorder often caused by mutations in the KIF7 gene, which disrupts the Sonic Hedgehog (SHH) signaling pathway, crucial for brain and limb development [15]. The pathophysiology results in agenesis or hypoplasia of the corpus callosum, craniofacial dysmorphism, polydactyly, and severe intellectual disabilities [15].

**Figure 2:**
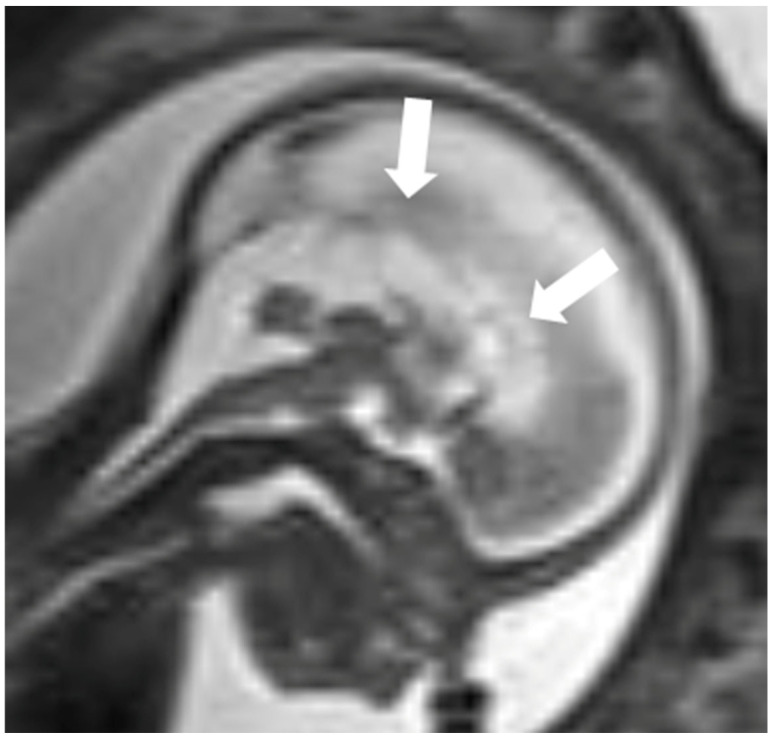
Fetal MRI sagittal T2W sequence at 21st week of gestation shows agenesis of corpus callosum (white arrow).

**Figure 3:**
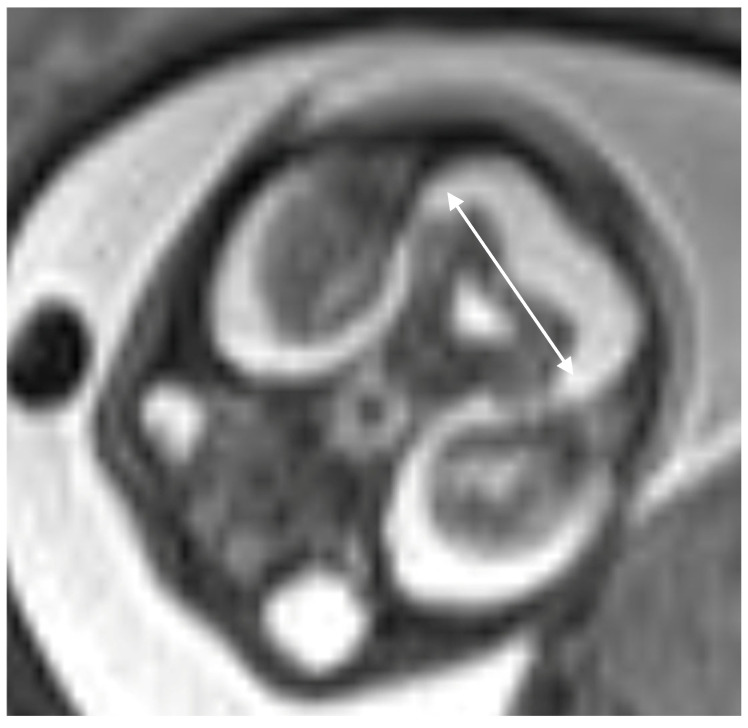
Fetal MRI axial T2W sequence at 21st week of gestation shows small cerebellum. Transcerebellar diameter is smaller than −3 SD (white double arrow).

Mutations in KIF7 disrupt SHH signaling, which is essential for patterning of the cortical midline and guiding callosal axons across it, resulting in agenesis or hypoplasia of the corpus callosum (Figure 2) [16,17]. SHH signaling is also critical for the proliferation of cerebellar granule neuron precursors (CGNPs) to facilitate proper development of the cerebellar architecture. Disruption of this signaling results in cerebellar hypoplasia (Figure 3) [18,19].

**Figure 4:**
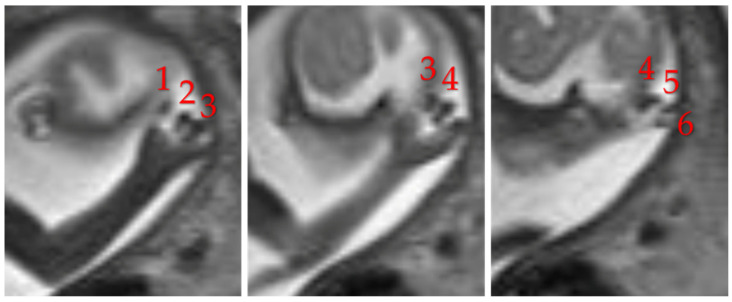
Fetal MRI T2W sequence at 21st week of gestation shows post-axial polydactyly (digits labeled with number).

In addition to intracranial abnormalities, the SHH pathway regulates the growth and patterning of the limb bud, including the formation of digits. Disruption of this pathway can lead to polydactyly (Figure 4) [16].

Fetal MRI can confirm the presence of corpus callosum agenesis or hypoplasia, interhemispheric cysts, and other associated brain abnormalities such as nodular cerebral cortex, hypoplastic pons and cerebellum, and neuronal heterotopias [20,21] Fetal MRI is also helpful for measurements of brain biometry using normative reference charts with difference measurements according to gestations, such as Kyriakopoulou et al. 2017 [22]. T2-weighted single-shot fast (turbo) spin-echo sequence is the preferred sequence to help evaluate the structural integrity of the brain, as it provides the highest tissue contrast between the gray matter, white matter zones, and cerebrospinal fluid [23]. These detailed imaging findings are crucial for accurate diagnosis and classification of the severity of ACS.

Fetal MRI plays a pivotal role in the multidisciplinary management of CNS genetic disorders. It provides high-resolution images that allow for detailed visualization of brain structures, aiding in the accurate diagnosis of malformations such as holoprosencephaly, lissencephaly, and agenesis of the corpus callosum. This imaging modality is particularly useful when ultrasound findings are inconclusive or when detailed anatomical information is required for prognosis and management planning [24,25].

Fetal MRI is integral to a multidisciplinary approach which includes genetic testing and parental counseling. This comprehensive approach ensures accurate diagnosis, informs prognosis, and guides decisions regarding pregnancy management and postnatal care [21,26].

### 2.2. Mitochondrial Disorders

Primary mitochondrial disorders constitute the most common cause of inborn errors of metabolism in children, and they frequently affect the central nervous system. Imaging findings of mitochondrial diseases in the prenatal period are less well described. In recent years, however, there have been increasing reports on fetal MRI features of pyruvate dehydrogenase complex deficiency (PDCD) [27,28,29,30,31,32]—a disorder of mitochondrial metabolism caused by pathogenic variants in various genes related to the pyruvate dehydrogenase complex (PDHc), a multiple enzyme complex that plays a critical role in mitochondrial energy production.

The PDHc converts pyruvate, the end product of glycolysis, to acetyl-CoA, which in turn enters the Krebs cycle to enable aerobic energy production. When PDHc cannot convert pyruvate to acetyl-CoA, excess pyruvate is instead converted into lactate, causing lactic acidosis and energy failure—often initial signs of PDCD. The brain is especially susceptible to this metabolic alteration. Therefore, PDCD predominantly affects the central nervous system.

Six genes are known to be associated with primary PDCD: PDHA1, PDHB, PDHX, DLAT, DLD, and PDP1, with PDHA1 being the most common, accounting for up to 90% of all known mutations. PDHA1 is X-linked, yet studies have found an equal number of males and females, with variable survival and disease course. PDHB, PDHX, DLAT, DLD, and PDP1 are autosomal recessive. In addition, PDCD also occurs secondary to conditions that impair PDC function, such as dietary thiamine deficiency and enzyme dysfunction upstream in the biochemical pathway, leading to deficiencies in cofactors essential for PDC function [33]. Examples of such genetic disorders include thiamine pyrophosphokinase deficiency (related to biallelic pathogenic variants in TPK1), mitochondrial short-chain enoyl-CoA hydratase 1 deficiency (related to biallelic pathogenic variants in ECHS1), and 3-hydroxyisobutyryl-CoA hydrolase deficiency (related to biallelic pathogenic variants in HIBCH) [33].

**Figure 5:**
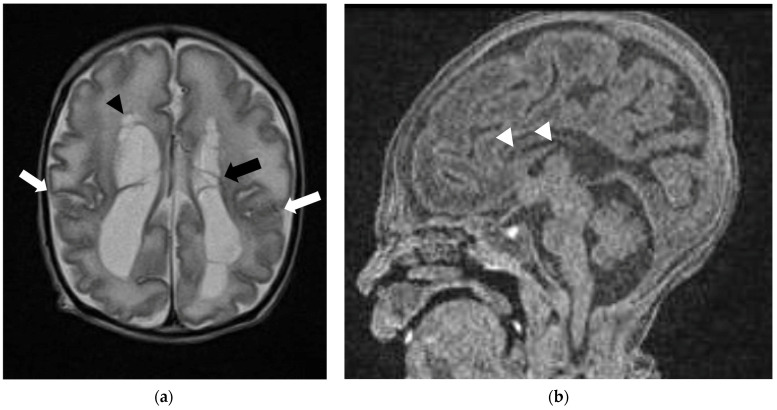
Neonate (38th week of gestation) presented with encephalopathy, subsequently confirmed PDCD with PDHA1 mutation. Antenatal ultrasound showed parenchymal cystic lesions, fetal MRI was not performed. Postnatal MRI performed on day 6 of life demonstrated findings characteristic of PDCD. (**a**) Axial T2W sequence shows germinolytic cysts (black arrow), periventricular cysts (black arrowhead), and gyration abnormality (white arrows); (**b**) sagittal T1W sequence at midline demonstrated short corpus callosum with absent posterior body, splenium, and rostrum, in keeping with callosal dysgenesis (white arrowheads).

Neuroimaging findings of PDCD are better known in the postnatal period, which include corpus callosum dysgenesis, cortical gyration abnormalities, and abnormalities involving the basal ganglia (Figure 5) [34,35]. Reported prenatal findings include corpus callosum dysgenesis, abnormal gyration pattern, reduced brain volumes, cystic lesions, intraventricular hemorrhages and midbrain malformation with aqueductal stenosis and severe hydrocephalus [27]. Therefore, recognition of characteristic fetal MRI findings can expedite genetic testing and enable earlier diagnosis.

## 3. Craniofacial Syndromes and Eye Syndromes

Craniofacial and ocular anomalies is an important group of structural abnormalities detectable on prenatal imaging, many of which have genetic causes and significant postnatal implications. Fetal MRI is supplementary to ultrasound by providing assessment of the cranial vault, facial structures, palate, orbits, and intracranial structures, particularly when sonographic evaluation is limited. Understanding the embryology, genetic basis and MRI features of these disorders is important for prenatal diagnosis, further genetic testing, prenatal parental counseling, and perinatal planning.

### 3.1. Craniofacial Syndrome

Craniofacial syndromes are a group of disorders characterized by malformation of the skull and facial bones.

#### 3.1.1. Malformations of Facial Bones

Cleft lip and/or palate is one of the most common congenital craniofacial defects resulting from incomplete fusion of the upper lip and/or palate. It occurs in approximately 1 in 1000 live births, with isolated cleft palate more common in females, whereas cleft lip with or without cleft palate is more frequent in males [36]. More than half (70%) of the cases are sporadic, while the remaining cases are syndromic or genetic, including trisomy 13 and 18, Treacher Collins syndrome, Van der Woude syndrome, etc. [37]. Embryologically, cleft lip results from disruption of the normal fusion between the nasal prominences and the maxillary prominences, while cleft palate arises from failure of fusion of the palatal shelves in the midline during the 6th to 8th week of gestation [38].

Clinically, infants present with visible discontinuity of the upper lip or palate. They may also have feeding difficulties and nasal regurgitation due to impaired palatal function [39]. Associated anomalies variably occur, particularly in syndromic forms, and may involve the craniofacial skeleton, eyes, ears, or other organ systems [40].

Fetal MRI plays an important complementary role in the prenatal assessment of cleft lip and palate, particularly when ultrasound assessment is limited by fetal position, maternal habitus, or oligohydramnios [41]. Cleft lip and palate can be detected on fetal MRI from 24th week of gestation onwards [42]. A meta-analysis including 334 fetuses demonstrated high diagnostic performance of fetal MRI for cleft palate, with pooled sensitivity of 97% (95% CI 0.93–0.99) and pooled specificity of 94% (95% CI 0.89–0.97) [43].

**Figure 6:**
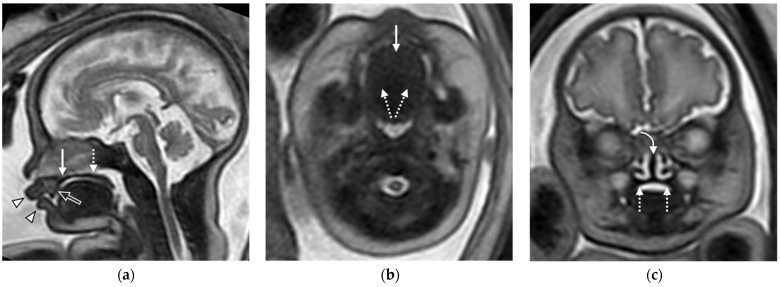
Fetal MRI of normal lip and palatal anatomy at 33rd week of gestation: (**a**) Sagittal T2-weighted image of the fetal head showing the intact upper and lower lips (arrowhead), primary palate (white arrow), secondary palate (dashed arrow), and alveolar ridge (open arrow); (**b**) axial T2-weighted image showing the intact primary palate (white arrow) fused with the bilateral secondary palates (dashed arrows); (**c**) coronal T2-weighted image showing the intact secondary palate (dashed arrows) fused with the nasal septum (curved arrow).

On fetal MRI, the primary and secondary palates, lip, and alveolus may be visualized (Figure 6) [41,44]. Clefts of the primary and secondary palates are best appreciated on axial and coronal views of the fetal face [41,45], which may be further characterized in terms of the cleft type, extent, and laterality. Additional MRI features include absence of tooth buds at a cleft alveolus and deviation of the nasal septum away from the side of the cleft [45]. In unilateral cleft lip and palate, fetal MRI may depict non-fusion of the hard palate with the nasal septum on the affected side [44]. Fetal MRI may also help pick up associated anomalies, including CNS abnormalities such as corpus callosal dysgenesis [46,47].

Clinically, prenatal diagnosis of cleft lip and/or palate is important for parental counseling, allowing early planning for neonatal feeding strategy and multidisciplinary care for postnatal surgical repair [48,49,50,51].

#### 3.1.2. Malformations of Skull Bones

Craniosynostosis refers to the premature fusion of one or more cranial sutures, restricting skull growth and producing characteristic calvarial deformities. The skull consists of the membranous calvarial vault, mainly neural crest-derived via intramembranous ossification, and the cartilaginous cranial base, mainly paraxial mesoderm-derived via endochondral ossification. They are joined by fibrous sutures and fontanelles that normally remain patent during gestation to allow brain growth [52].

The prevalence of craniosynostosis was estimated to be approximately 1 in 2500 births [53], with at least 20% attributable to an underlying genetic disorder [54]. The most commonly implicated genes include FGFR1, FGFR2, FGFR3, TWIST1, and MSX2 [55]. Among these, FGFR mutations are the genetic basis of Crouzon and Apert syndromes, which are discussed below.

Crouzon syndrome is an autosomal dominant craniosynostosis caused primarily by FGFR2 mutations (10q26), occurring in 1 in 25,000 births [56]. FGFR gain-of-function mutations accelerate osteoblastic differentiation and thus suture ossification [57]. The coronal sutures are most commonly involved, with compensatory growth at unfused sutures producing brachycephaly or, in severe multisutural cases, cloverleaf skull [58]. Resultant anterior cranial base deformities cause shallow orbits, midface hypoplasia, and maxillary underdevelopment. Clinically, children may present with the triad of calvarial deformity, midface hypoplasia, and proptosis [59], sometimes with dental malocclusion or upper airway obstruction [60]. Limb anomalies are typically absent, distinguishing Crouzon from Apert syndrome [56].

Apert syndrome is an FGFR2-related craniosynostosis disorder, with a prevalence of 1 in 65,000 births (4.5% of craniosynostosis cases) [61]. Most cases arise from de novo FGFR2 mutations. Like Crouzon syndrome, it involves FGFR2 gain-of-function mutations but usually causes a more severe craniofacial phenotype [62]. Clinically, an affected child may present with the triad of craniosynostosis, midface hypoplasia, and symmetrical syndactyly of hands and feet. Bilateral coronal sutures are commonly involved with metopic widening, while facial features may include hypertelorism, flat nasal bridge, and cleft palate. When compared to Crouzon syndrome, Apert syndrome tends to show more severe and asymmetrical craniofacial dysmorphism, thus exhibiting greater functional and esthetic impact [62,63].

The role of fetal MRI in the assessment of craniosynostosis remains limited, which was mainly described in case reports [64,65,66] and small retrospective series [67,68,69,70]. A primary limitation is that calvarial sutures cannot be directly visualized on fetal MRI, hence diagnosis relies on detection of abnormal skull morphology as a secondary sign of sutural fusion [71]. In addition, as cranial sutures only form at 15th–16th week of gestation, imaging diagnosis will be challenging in the first and early second trimesters [52]. Nonetheless, fetal MRI remains valuable for evaluating associated manifestations of craniosynostosis, including craniofacial abnormalities involving the palate, mandible, and orbits [72], intracranial abnormalities such as hydrocephalus and midline anomalies, as well as limb deformities [73].

Prenatal diagnosis of craniosynostosis is important for parental counseling and postnatal management. Once a craniosynostosis syndrome is suspected, fetal genetic evaluation, including chromosomal microarray analysis on amniotic fluid cells and targeted molecular testing, may be considered for genetic counseling [74]. When severe craniofacial deformities are present, postnatal craniofacial surgery such as craniotomy and midface advancement may be required. Early identification of midface hypoplasia is particularly important, as it predisposes the neonate to upper airway obstruction, which requires advanced planning for potential neonatal airway interventions such as intubation [75]. Fetal MRI is therefore important for multidisciplinary postnatal care.

### 3.2. Eye Syndromes

The eyes develop from paired optic vesicles, which emerge from the forebrain at around the 3rd week of gestation and induce formation of the lens placode upon contact with the surface ectoderm [76]. Subsequent infolding of the optic vesicle forms the optic cup and the developing lens. By five to seven weeks, the lens detaches from the surface ectoderm, and the globe gradually forms as the optic cup expands. The hyaloid artery, which initially supplies the lens, regresses after the tenth week, leaving a remnant known as the Cloquet canal [76,77].

The anophthalmia–microphthalmia spectrum is among the more common genetically associated ocular abnormalities detectable on fetal MRI, with a reported prevalence of 2.5–3.2 per 10,000 births [78,79]. Anophthalmia is defined as complete absence of the globe, with preservation of periocular structures such as the eyelids, conjunctiva, and lacrimal apparatus, whereas microphthalmia refers to a markedly reduced globe size [76]. Primary anophthalmia results from failure of early ocular development and is frequently associated with chromosomal or single-gene disorders, including trisomy 13, SOX2-related disease, Walker–Warburg syndrome, and CHARGE syndrome [80]. Secondary anophthalmia arises following an in utero insult occurring after normal early-eye formation, such as infection, toxin, or metabolic disturbance [76].

**Figure 7:**
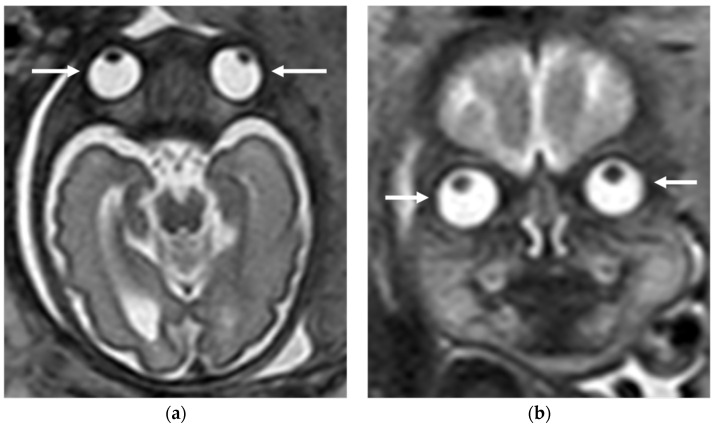
Fetal MRI of normal globes at 29th week of gestation: (**a**) Axial T2-weighted image and (**b**) coronal T2-weighted image showing normally formed bilateral symmetrical globes (white arrows).

Fetal MRI for congenital eye disorders may be performed following concerning sonographic findings of the orbits or when the globes are not clearly visualized on a mid-gestation morphology scan [81]. Fetal MRI allows assessment of the globes (Figure 7), orbital biometry using MRI-specific growth curves, and demonstrates an absent globe or markedly reduced globe size [82]. In addition, fetal MRI can identify associated intracranial malformations—such as midline abnormalities—which may indicate underlying syndromic disease and guide further management, including targeted genetic testing, prenatal parental counseling, and postnatal care planning [70].

### 3.3. Conclusions

Fetal MRI is valuable in the assessment of genetic disorders with craniofacial and ocular abnormalities, helping prenatal diagnosis and detection of associated anomalies. It may guide further genetic testing, aiding in prenatal parental counseling and perinatal management.

## 4. Skeletal Dysplasia

Skeletal dysplasia is a vast heterogeneous group of genetic diseases with widespread abnormalities in the skeleton. Development of bone and cartilage is affected, resulting in various skeletal phenotypes. According to the latest nosology of genetic skeletal disorders revised in 2023, there are currently 771 entities in this group, associated with 552 identified genes [83]. Classification framework of skeletal dysplasia is complex, relating to an evolving understanding that is rapidly expanding but not yet thorough. These entities are classified into 41 different groups, according to their genetic basis, pathogenesis, morphological description, or regions affected. While individual entities within this group are rare, they are collectively not that uncommon. As a group, skeletal dysplasia affects around 1 in 3000–5000 births [84,85]. Thanks to the wide availability of next-generation sequencing techniques, identification of causative genes and the understanding of molecular basis in skeletal dysplasia have rapidly increased in the last 2 decades [86,87]. Accurate diagnosis made earlier in the prenatal period is beneficial in clinical management and parental counseling. Skeletal dysplasia with intrauterine development of disproportionate limb shortening (e.g., Achondroplasia, Achondrogenesis), abnormal bone shape (e.g., osteogenesis imperfecta due to fractures, campomelic dysplasia with bowing), and thoracic hypoplasia (e.g., thanatophoric dysplasia) can potentially be diagnosed prenatally. This section explores the role of fetal MRI as an adjunct in evaluation of fetal skeletal dysplasia.

Second trimester ultrasound scan, being a part of standard of care in prenatal check-up in many countries [88], is the first screening tool for fetal skeletal dysplasia [84,89]. Fetal skeleton can be visualized sonographically by 14th week of gestation, allowing measurements of fetal long bones in the second-trimester structural scan [89]. Assessment of biometry of long bones, plus assessment of parameters such as head circumference and femur-to-foot ratio, detected disproportionation in skeletal dysplasia [89,90,91]. While ultrasound remains the primary screening tool, it is not without limitations. Large maternal body build, oligohydramnios (which can be associated with some skeletal dysplasia), and suboptimal fetal positioning can render visualization by ultrasound limited [84,91]. Ultrasound provides little information about mineralization [91]. Also, some skeletal dysplasia may not present itself until late into the third trimester, making ultrasound assessment challenging [84,91].

**Figure 8:**
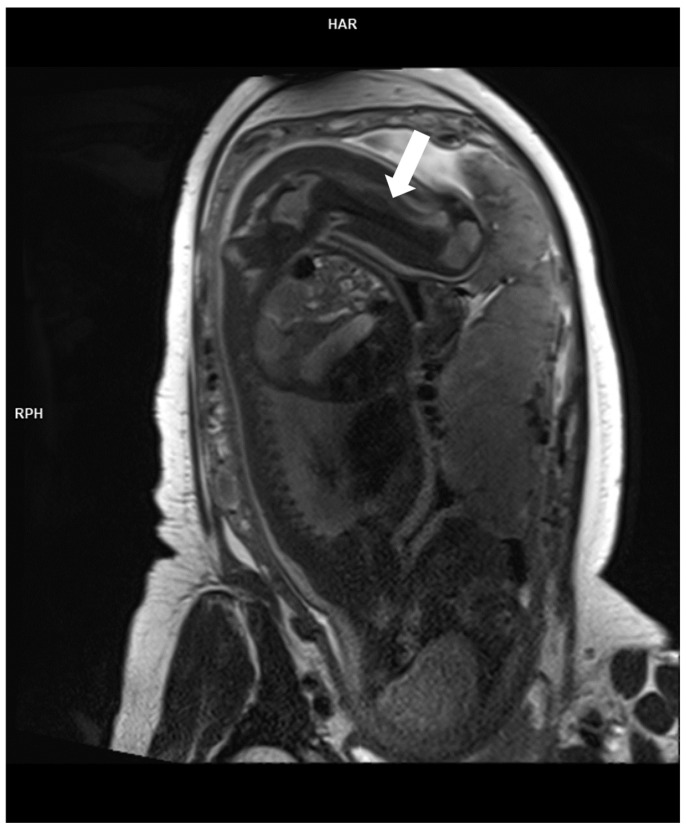
T2W HASTE image of fetal MRI at 34th week of gestation with femur (white arrow) imaged in profile. This image demonstrates the excellent contrast resolution of fetal MRI, enabling evaluation of morphology, mineralization, and cartilaginous components of the skeleton.

Fetal MRI has the advantage of having a larger field of view and good contrast resolution with possibility of 3D reconstruction [84]. Abnormal long bone morphologies, such as bowing, can be readily seen on fetal MRI scans. Abnormalities in epiphyseal cartilage, seen in many skeletal dysplasia such as type II collagen disorders and chondrodysplasia punctata, can be depicted on fetal MRI as abnormal cartilage signals [84,92,93]. Figure 8 shows a T2W HASTE image of fetal MRI with femur imaged in profile, clearly depicts its morphology and allows accurate measurement. Mineralization and cartilage abnormalities can be assessed by altered signal on fetal MRI. Dovjak et al. reported a suspected case of chondrodysplasia punctata with inconclusive prenatal USG findings. Fetal MRI showed low signal in bones on echo planar imaging signifying premature calcification and epiphyseal calcifications, confirming the diagnosis and facilitated decision of termination of pregnancy [93]. In a pre-clinical study, a novel 3T MRI technique using T2*-weighted multiple gradient-echo sequence with specific TE and flip-angle has been shown to increase contrast between bone and soft tissue, being reported to be superior than CT scan in visualization of fetal skeleton, including small bones such as metacarpals and metatarsals [94].

Some skeletal dysplasias are associated with central nervous system and spinal abnormalities, which are better assessed by fetal MRI [84,89]. Thanatophoric dysplasia is an entity with severe limb shortening, narrow chest, and abnormal calvarial contour. Affected fetuses also show characteristic abnormal gyration and deep sulcation in temporal lobe, best visualized by fetal MRI [84,95]. Fetal MRI can increase the confidence in diagnosing this uniformly lethal condition, helpful in making critical decisions such as termination of pregnancy [84].

**Figure 9:**
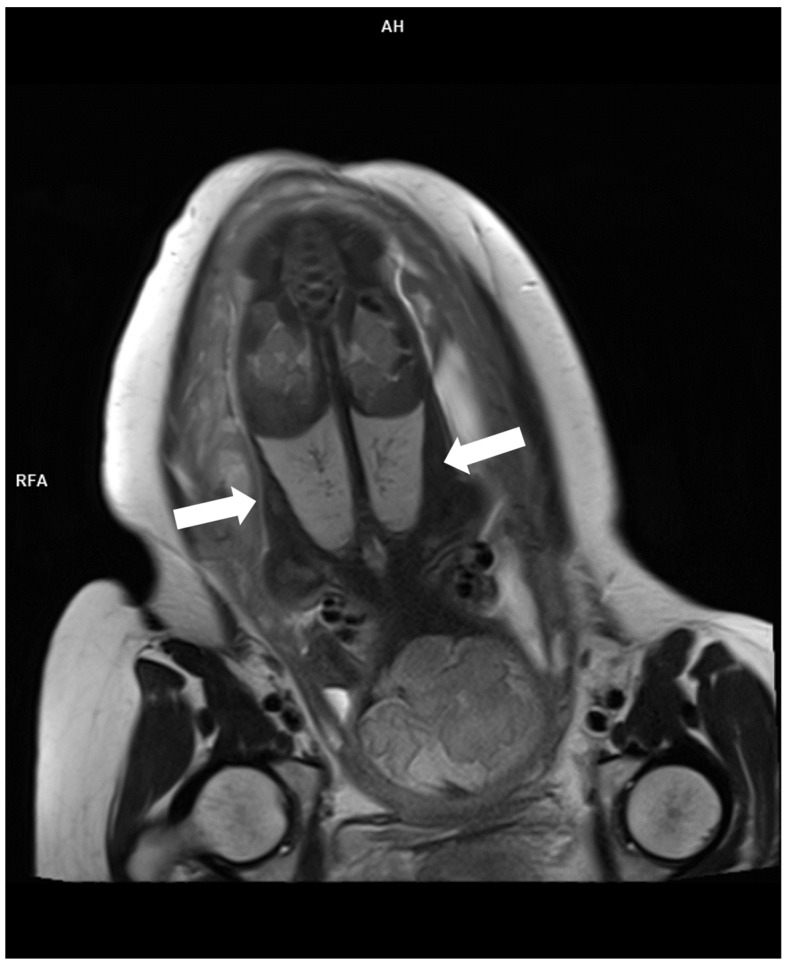
T2W HASTE image of fetal MRI at 32nd week of gestation clearly depicting the lungs (white arrows), enabling segmentation and accurate estimation of lung volume, helpful in predicting lethality of skeletal dysplasia.

Achondroplasia, the most common skeletal dysplasia, is associated with craniocervical stenosis, abnormal sulcation of temporal lobe, incomplete hippocampal rotation, and spinal abnormalities such as thoracolumbar kyphosis [84,96]. Fetal MRI not only provides superior visualization of these to raise diagnostic accuracy, but it also allows detailed evaluation to help in planning delivery and perinatal care [84,89]. Pulmonary hypoplasia is the primary cause of lethality in fetal skeletal dysplasia [84,97]. Fetal MRI provides outstanding visualization of fetal lungs (Figure 9), enabling segmentation for accurate lung volume assessment [98]. This provides crucial information to predict lethality, as well as derive delivery and perinatal resuscitation plan when sonographic assessment of lung volume is limited [84,89,97].

Entities of skeletal dysplasia share overlapping features and diagnosis in the prenatal period is challenging. Fetal MRI is a useful adjunct and is complementary to prenatal ultrasound scan for detailed fetal phenotyping [84,89,99], crucial in initiating molecular testing. Accurate phenotyping is also indispensable in the interpretation of molecular testing results to land on an accurate diagnosis. Fetal MRI’s role in the multidisciplinary management of fetal skeletal dysplasia should be recognized and emphasized.

## 5. Congenital Lung Malformations

Lungs are a complex organ system with network of branched airways, blood vessels and alveoli, forming a structure that enables gas exchange [100]. The development of lungs is also complex.

During lung development, the conducting airways are formed first, followed by formation and enlargement of the gas exchange area until young adulthood [101]. It begins at 4th week of gestation and is divided into five different stages based on morphological criteria including embryonic, pseudoglandular, canalicular, saccular, and alveolar stages [100,101,102,103]. The timing of the stages is said to be overlapping due to non-synchronous lung development, with most processes starting proximally and extending into the periphery [101,102].

The embryonic stage (from 4th to 7th week of gestation) starts with separation the trachea from the foregut. Two lung buds appear as outpouching of respiratory epithelial cells on ventral part of the foregut towards end of 4th week. In this stage, key genes are expressed in endoderm (Nkx21 encoding TTF1 in ventral wall of anterior foregut and Sox2 and Hoxb5 in dorsal wall of anterior foregut) and surrounding mesenchyme (BMP4 and its antagonist Noggin, FGF10 and Wnt). Lung buds then extend and separate into branches, creating primitive bronchi. Pulmonary arteries develop from the sixth aortic arches and grow into the mesenchyme to form a vascular plexus. During the pseudoglandular stage (from 8th to 16th week of gestation), branching morphogenesis occurs to form bronchial tree down to preacinar airways. The pattern depends on FGF10 expression in the distal mesoderm through its receptor fibroblast growth factor (FGFR2b) in adjacent epithelium for bud growth with negative feedback loops by SPRY2, SHH, TGFβ, and Wnt pathways inhibiting bud outgrowth. In the canalicular stage (from 17th to 24th week of gestation), branching morphogenesis continues with formation of acinus and blood–air interface is prepared with capillaries leaning against the epithelium. Pulmonary epithelium continues to differentiate into type II and type I pneumocytes. In saccular stage (from 25th to 35th week of gestation), saccules form at terminal airways with widening of peripheral air spaces. Surfactant production starts into primitive alveoli at around 26th week. This stage marks the earliest period of lung viability. Lung development continues as alveolar stage which begins after birth and continues until 4–5 years old, with establishment of secondary septa subdividing saccules into alveoli to increase gas exchange surface [102,103,104,105,106].

Congenital lung malformations (CLMs) account for up to 18% of all congenital abnormalities [103,107]. They include a broad spectrum of diseases, with the more common ones being congenital pulmonary airway malformation (CPAM), bronchopulmonary sequestration (BPS), congenital lobar overinflation (CLO), and bronchogenic cyst [103]. Overall, it is relatively rare with reported incidence of 1 in 15,000 live births, although true incidence is likely higher with inclusion of stillbirths and abortions as well as increased use and advances in imaging [108,109].

The etiology of CLM is controversial, with several hypotheses proposed. A widely proposed one is airway malformations being related to embryonic insults, with types of specific lesion related to level and degree of obstruction and timing of the insult. This hypothesis might explain the overlapping features in different malformations [107,109]. On the other hand, genetic research in animal study and resected tissue of the malformations have shown possible links between CLMs and genes regulating lung development [103,105,107]. For example, acinar dysplasia, which is the preferred term for CPAM type 0, is associated with mutations in genes encoding FGF10, FGFR2, and the transcription factor TBX4 [103]. These results raised concerns about possibility of genetic basis of CLM and may provide new development in diagnosis and management of CLM.

**Figure 10:**
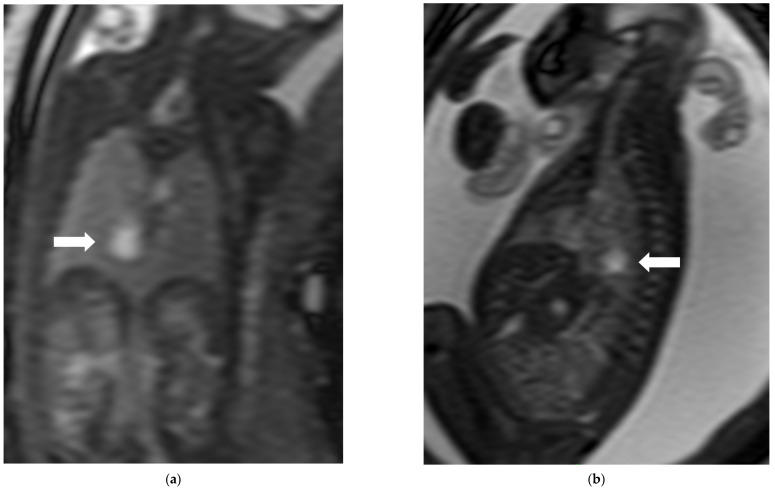
Fetal MRI was performed at 23rd week of gestation for delineation of hyperechoic mass with cystic components in mediastinum. (**a**) Coronal T2W sequence and (**b**) sagittal T2W sequence shows a T2W hyperintense cystic lesion in the medial aspect of the right lung adjacent to mediastinum, suggestive of congenital pulmonary airway malformation (CPAM).

The two major indications of fetal MRI for thoracic conditions are CLM and congenital diaphragmatic hernia [8,10]. Fetal MRI is highly accurate in diagnosing specific congenital lung lesions (98%) by evaluating signal intensity, feeding vessels, number of cysts, and presence of architectural distortion of the lesions [110]. It can also evaluate size of the lesions and their mass effects on surrounding structures such as compression of venous return to the heart, resulting in right-sided heart failure and hydrops [111]. In addition to characterization of lung lesions, lung volume measurement by fetal MRI is used as a surrogate to reflect degree of pulmonary hypoplasia in various thoracic and extra-thoracic conditions to predict postnatal outcome such as survival rates in congenital diaphragmatic hernia, omphalocoele, congenital high airway obstruction, and genitourinary abnormalities and oligohydramnios [112]. Standard fetal MRI sequences of lungs mainly include fluid-sensitive sequences such as half-Fourier single-shot turbo spin-echo (SSTSE) and free induction steady-state (SSFP), T1-weighted fast low-angle shot (FLASH), and echo planar imaging [7,111]. Diagnostic accuracy of fetal MRI may be limited by small lung lesion in the fetus. Figure 10 shows a T2W image of a T2W hyperintense cystic lesion in the right lung, suggestive of CPAM with bronchogenic cyst as differential diagnosis. Therefore, an antenatal diagnostic approach of CLM usually only includes serial fetal USG for close monitoring, while fetal MRI is recommended when a lung lesion is not clearly delineated in fetal USG or for volumetric assessment of a large CLM and fetal lung parenchyma [8,10,103].

## 6. Urinary System Genetic Disorders

Congenital Anomalies of the Kidney and Urinary Tract (CAKUT) represent one of the most frequently encountered categories of genetic disorders in prenatal diagnosis, accounting for approximately 20–30% of prenatally detected structural malformations [113]. While mild urinary tract dilatation is often physiologic and self-limiting, significant genitourinary anomalies—including polycystic kidney disease, renal agenesis or dysplasia, and bladder outlet obstruction—require comprehensive prenatal evaluation, genetic testing, and multidisciplinary counseling to optimize perinatal management.

Within CAKUT, urinary anomalies can be broadly categorized into (1) cystic kidney diseases (e.g., ARPKD, ADPKD, syndromic ciliopathies), (2) renal agenesis and hypodysplasia/dysplasia, and (3) lower urinary tract obstruction (LUTO) and megacystis phenotypes. In this section, we use the following abbreviations: CAKUT (congenital anomalies of the kidney and urinary tract), ARPKD (autosomal recessive polycystic kidney disease), ADPKD (autosomal dominant polycystic kidney disease), MCDK (multicystic dysplastic kidney), LUTO (lower urinary tract obstruction), PUV (posterior urethral valves), and MMIHS (megacystis–microcolon–intestinal hypoperistalsis syndrome).

Fetal MRI has evolved from a complementary imaging tool to an indispensable modality when ultrasound is non-diagnostic or when precise anatomical and tissue characterization is required for definitive diagnosis, prognostication, or consideration of fetal intervention [114]. Its strength lies in its multiplanar imaging capability, superior soft-tissue contrast independent of amniotic fluid volume, and freedom from acoustic shadowing by maternal habitus or fetal skeletal ossification, particularly in the third trimester [115]. Fetal MRI excels in differentiating cystic kidney diseases, confirming the presence or absence of renal parenchyma, delineating complex pelvic anatomy, and assessing secondary consequences such as oligohydramnios-related pulmonary hypoplasia [115]. These imaging findings carry significant prognostic weight and directly inform perinatal care planning, parental counseling, and the choice of genetic testing strategy [116].

In small retrospective series of fetuses with suspected renal anomalies, fetal MRI has been shown to refine or change the ultrasound diagnosis in roughly one-third of cases and to achieve sensitivities in the range of 85–90% for distinguishing cystic kidney disease from renal agenesis when compared with postnatal imaging or autopsy [114,116].

### 6.1. Cystic Kidney Disease

Autosomal Recessive Polycystic Kidney Disease (ARPKD) is caused by biallelic mutations in the PKHD1 gene encoding fibrocystin, a protein critical for ciliary function and epithelial differentiation. The classic sonographic appearance consists of bilaterally enlarged, markedly hyperechogenic kidneys with preserved reniform contour. Fetal MRI provides distinctive tissue characterization, demonstrating massively enlarged kidneys with diffuse T2 hyperintensity that reflects the water content within innumerable microcysts (ectasia of collecting ducts) that are below the spatial resolution of ultrasound. A key diagnostic feature is the absence of macroscopic discrete cysts and the lack of intervening normal parenchyma, which helps differentiate ARPKD from autosomal dominant forms and other cystic dysplasias [117]. While congenital hepatic fibrosis—a defining feature of ARPKD—is challenging to visualize directly in utero, fetal MRI may occasionally identify biliary ductal ectasia or macroscopic hepatic cysts in severe cases, supporting the diagnosis.

Autosomal Dominant Polycystic Kidney Disease (ADPKD) results from mutations in PKD1 (approximately 85% of cases) or PKD2 (approximately 15%). Although typically an adult-onset disorder, severe phenotypes may manifest prenatally with enlarged, hyperechogenic kidneys mimicking ARPKD. Fetal MRI is more sensitive than ultrasound for detecting discrete, macroscopic cysts distributed within both the cortex and medulla, a finding that strongly favors ADPKD over ARPKD (Table 1) [113]. In small retrospective series of fetal cystic kidney disease, MRI detects more and smaller cortical cysts than ultrasound and improves subclassification along the ARPKD–ADPKD–MCDK spectrum [113]. Crucially, prenatal detection should prompt parental renal imaging; identification of asymptomatic cysts in a parent supports autosomal dominant inheritance (50% recurrence risk) rather than autosomal recessive (25%), with major implications for counseling.

Other genetically transmitted renal cystic diseases include Bardet–Biedl syndrome and Meckel–Gruber syndrome. These ciliopathies often present with enlarged, dysplastic kidneys containing multiple cysts and associated extra-renal features—such as polydactyly, occipital encephalocele, or hepatic fibrosis—that are well depicted on MRI and guide targeted testing of ciliary gene panels.

From a genetic-testing standpoint, fetuses with bilaterally enlarged echogenic kidneys and diffuse microcystic pattern are generally offered chromosomal microarray followed by targeted PKHD1 sequencing; when macrocysts are seen and a parent has renal cysts, PKD1/PKD2 testing is prioritized. In suspected syndromic cystic kidneys with polydactyly or CNS malformations, ciliopathy gene panels (e.g., TMEM67, MKS1, BBS genes) are indicated [117].

### 6.2. Renal Agenesis

**Figure 11:**
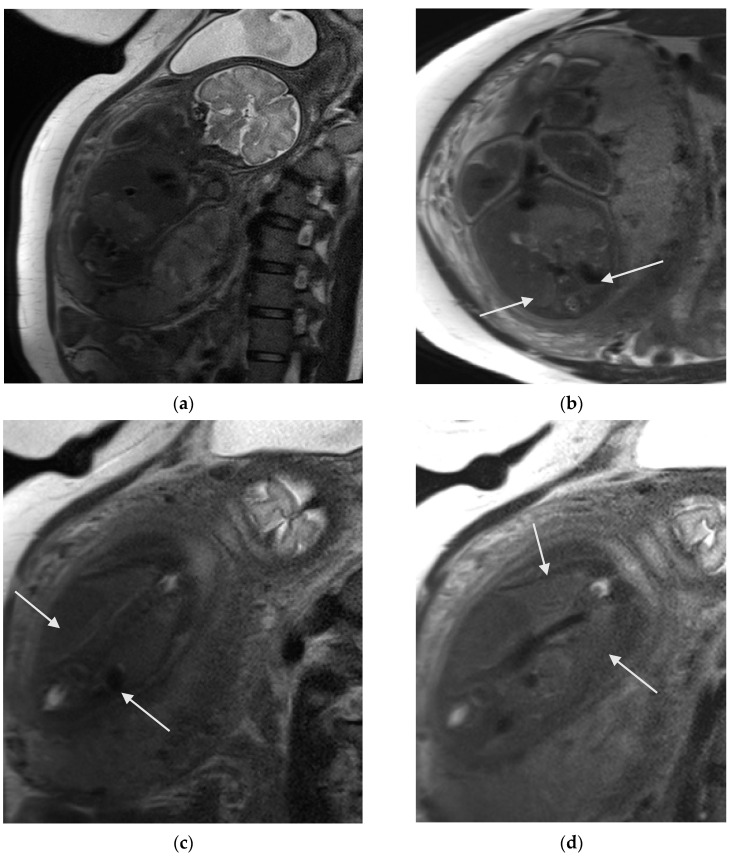
Fetal MRI at 32th week of gestation with bilateral renal agenesis. (**a**) Coronal T2W HASTE image shows severe anhydraminios. (**b**) Axial and (**c**) coronal T2W HASTE images show that bilateral renal fossae (white arrows) are empty, with no reniform structure identified. No fluid-filled structure in pelvis to suggest urinary bladder. (**d**) Coronal T2W HASTE image shows bell-shaped thorax (white arrows), suggestive of pulmonary hypoplasia.

Bilateral renal agenesis is a lethal condition characterized by complete absence of renal tissue, resulting in anhydramnios and pulmonary hypoplasia (Potter sequence). Diagnosis on ultrasound due to severe oligohydramnios and the potential for adrenal glands to mimic kidneys. Fetal MRI is considered the definitive modality in equivocal cases, demonstrating empty renal fossae, absence of reniform structures in both abdomen and pelvis, and elongated “lying-down” adrenal glands (Figure 11). MRI also allows systematic search for associated anomalies suggestive of syndromic etiologies (e.g., Fraser syndrome, VACTERL association).

A key strength of MRI in this context is quantitative assessment of lung volume. Observed-to-expected (O/E) total fetal lung volume derived from T2-weighted sequences provides an objective surrogate for pulmonary hypoplasia. Cohort studies in fetuses with bilateral renal agenesis or severe CAKUT report that an O/E lung volume below approximately 25–30% is strongly associated with non-survival despite intensive postnatal support [97,112]. Including this metric in multidisciplinary discussions is valuable for counseling about prognosis and the likely futility of aggressive resuscitation.

Unilateral renal agenesis, which occurs in around 1 in 1000 live births, is typically compatible with normal postnatal life. Accurate fetal MRI differentiation directly impacts decisions regarding pregnancy continuation, genetic testing strategy, and family planning.

### 6.3. Renal Dysplasia

Renal dysplasia denotes abnormal tissue differentiation with disorganized parenchymal architecture. On fetal MRI, dysplastic kidneys typically show increased T2 signal intensity relative to liver or muscle, poor corticomedullary differentiation (CMD), and sometimes small cortical or subcortical cysts. Several studies have shown that more severe loss of CMD and marked T2 hyperintensity correlate with reduced postnatal renal function [76].

A “reversed CMD” pattern—T2-hyperintense cortex with relatively hypointense medulla—has been described most consistently in fetuses with 17q12 deletions involving HNF1B [113]. This pattern is highly suggestive but not absolutely specific for 17q12 deletion; similar appearances can rarely occur in other hypodysplasia syndromes. In practice, reversed CMD or striking CMD abnormalities in bilaterally small or echogenic kidneys should trigger targeted testing for HNF1B first, followed by broader CAKUT gene panels if negative.

Monogenic CAKUT should be particularly suspected when any of the following are present: bilateral hypoplastic or dysplastic kidneys (with or without cysts), reversed or markedly abnormal CMD, CAKUT combined with extra-renal anomalies (e.g., diabetes or pancreatic changes suggesting HNF1B disease; branchial cleft or ear anomalies suggesting EYA1/SIX1/SIX2-related branchio-oto-renal spectrum; polydactyly or encephalocele suggesting ciliopathy), or a strong family history of renal malformations or early-onset chronic kidney disease [113].

Multicystic Dysplastic Kidney (MCDK) is characterized by a non-functioning kidney replaced by multiple non-communicating cysts of varying sizes with absent normal parenchyma. On fetal MRI, the affected kidney appears as a cluster of T2-hyperintense cysts with no visible renal cortex or medulla. Cohort studies of prenatally diagnosed unilateral MCDK report contralateral renal or urinary tract anomalies—most commonly vesicoureteral reflux or ureteropelvic junction obstruction—in approximately 20–30% of cases [113]. Accordingly, careful MRI evaluation of the contralateral kidney and urinary tract is essential, and postnatal surveillance is warranted even when the opposite kidney appears structurally normal in utero.

### 6.4. Lower Urinary Tract Obstruction (LUTO)

Posterior urethral valves are the most common cause of congenital bladder outlet obstruction in male fetuses. On imaging, the classic “keyhole sign” represents a dilated bladder and posterior urethra. Fetal MRI is particularly helpful when ultrasound is limited by maternal habitus or oligohydramnios and in differentiating PUV from urethral atresia or non-obstructive causes of megacystis. MRI depicts bladder size and wall thickness, proximal urethral configuration, degree of upper-tract dilatation, and parenchymal signal changes in the kidneys.

From a practical perspective, MRI plays a central role in selecting candidates for fetal intervention. Fetuses with LUTO are evaluated for (1) evidence of true mechanical obstruction (dilated posterior urethra, thick-walled bladder), (2) degree of renal damage (cortical thinning, increased T2 signal, CMD loss), and (3) severity of pulmonary hypoplasia (O/E lung volume). Intervention (e.g., vesico-amniotic shunting or fetoscopic valve ablation) is selected based on presence of mechanical obstruction and severity of renal damage/pulmonary hypoplasia [118,119].

In contrast, megacystis–microcolon–intestinal hypoperistalsis syndrome (MMIHS) is a primary visceral myopathy caused by pathogenic variants in ACTG2, MYH11, LMOD1, and related smooth-muscle genes. MRI shows a massively dilated bladder without the keyhole configuration, a narrow-caliber unused colon, and dilated small-bowel loops with absent peristalsis. Amniotic fluid volume is usually normal or increased (polyhydramnios) due to impaired swallowing and intestinal dysmotility rather than oliguria. Recognizing this pattern is crucial, as these fetuses are not candidates for urological decompression and instead require postnatal surgical and gastroenterological planning.

Prognostically, MRI markers associated with poor outcome in LUTO include very thin and bilateral T2-hyperintense renal cortex, absent corticomedullary differentiation, evidence of renal dysplasia, severe bilateral hydroureteronephrosis, and markedly reduced O/E lung volume. In contrast, unilateral obstruction or preserved cortical thickness with near-normal lung volume is associated with better survival and the possibility of meaningful renal function in at least one kidney [119].

### 6.5. Conclusions

Fetal MRI has become an indispensable component of the diagnostic algorithm for genetic urinary system disorders. By overcoming the limitations of ultrasound in severe oligohydramnios and providing detailed tissue characterization, fetal MRI enhances diagnostic precision for conditions such as polycystic kidney disease, renal agenesis, dysplasia, and LUTO [7]. Fetal MRI findings stratify disease severity, guide selection of targeted genetic testing based on imaging phenotype, and inform complex decisions regarding fetal intervention and perinatal care strategies.

## 7. Fetal Proportional Analysis

In addition to structural evaluation, fetal proportional analysis may provide a useful adjunct in the assessment of suspected chromosomal abnormalities. A study of 145 chromosomally abnormal fetuses by Falcon O et al. demonstrated that the fetal head-to-trunk volume ratio during measured during 11th to 13th week of gestation using three-dimensional (3D) ultrasound was significantly increased in trisomy 13, trisomy 18, and triploidy (*p* < 0.001). In addition, the relative fetal trunk volume was significantly reduced in all chromosomal abnormalities (*p* < 0.001), except in Turner syndrome [120]. These measurements may serve as imaging markers of underlying chromosomal abnormalities. Although the measurements are more commonly performed using ultrasound, potential use of regional volumetric evaluation by fetal MRI may also be feasible in selected cases.

## 8. Conclusions

This review summarized the application of fetal MRI for genetic disorders in multiple organ systems and its roles in multidisciplinary team management. This narrative review has some limitations. Our literature search method only included several most popular search databases with a long search period. Many radiological references are case reports and small case series. The case examples illustrated in this review are representative yet very limited. Our review has not included updated fetal MRI protocols, fetal MRI safety and technical considerations, and use of artificial intelligence in where major advancements in technology lie. A future systematic review should include a wider range of search databases with emphasis on fetal MRI techniques and recommendations in management.

Familiarizing embryology and genetic basis of different genetic disorders is essential for interpretation of fetal MRI. On the other hand, when genetic disorders are suspected or confirmed, fetal MRI added values in guiding further genetic testing, complementing ultrasound, aiding prenatal diagnosis, and providing necessary information for prenatal and postnatal management and parental counseling.

Given the advancements in both genetic testing and fetal MRI, possible diagnostic pathways of genetic disorders can start with abnormal prenatal ultrasound findings followed by fetal MRI when indicated. The fetal MRI phenotypes can guide targeted genetic panel testing. This integrated approach will further improve perinatal diagnosis and management of genetic disorders in the era of precision medicine.

## Figures and Tables

**Table 1:** ARPKD versus ADPKD: genetics and fetal MRI phenotype.

Feature	ARPKD	ADPKD
Inheritance	Autosomal recessive	Autosomal dominant
Main gene(s)	PKHD1	PKD1, PKD2
Typical gestation for detection	Mid–late 2nd trimester	Late 2nd–3rd trimester
Kidney size	Markedly enlarged, smooth contour	Enlarged, sometimes asymmetric
Parenchymal signal	Diffuse T2 hyperintensity with fine microcystic texture	Heterogeneous parenchyma with discrete macrocysts in cortex/medulla
Macroscopic cysts on MRI	Absent or minimal	Common and often numerous
Hepatic findings	Possible biliary ectasia, congenital hepatic fibrosis	Usually absent prenatally

## Data Availability

No new data were created in this study.
